# A novel basement membrane-related gene signature predicts prognosis and immunotherapy response in hepatocellular carcinoma

**DOI:** 10.3389/fonc.2024.1388016

**Published:** 2024-07-12

**Authors:** Bingyao Li, Yingkun Che, Puhua Zhu, Yuanpeng Xu, Haibo Yu, Deyu Li, Xiangming Ding

**Affiliations:** ^1^ Henan Provincial People’s Hospital, Xinxiang Medical University, Xinxiang, Henan, China; ^2^ Henan Provincial Key Medical Laboratory for Hepatobiliary and Pancreatic Diseases, Henan Provincial People’s Hospital, Zhengzhou, Henan, China; ^3^ Department of Hepatobiliary Pancreatic Surgery, People’s Hospital of Zhengzhou University, Henan Provincial People’s Hospital, Zhengzhou, Henan, China; ^4^ Department of Gastroenterology, People’s Hospital of Zhengzhou University, Henan Provincial People’s Hospital, Zhengzhou, Henan, China; ^5^ Henan Key Medical Laboratory for Molecular Immunology of Digestive Diseases, Henan Provincial People’s Hospital, Zhengzhou, Henan, China

**Keywords:** basement membrane, hepatocellular carcinoma, tumor microenvironment, prognosis, immunotherapy

## Abstract

**Background:**

Basement membranes (BMs) have recently emerged as significant players in cancer progression and metastasis, rendering them promising targets for potential anti-cancer therapies. Here, we aimed to develop a novel signature of basement membrane-related genes (BMRGs) for the prediction of clinical prognosis and tumor microenvironment in hepatocellular carcinoma (HCC).

**Methods:**

The differentially expressed BMRGs were subjected to univariate Cox regression analysis to identify BMRGs with prognostic significance. A six-BMRGs risk score model was constructed using Least Absolute Shrinkage Selection Operator (LASSO) Cox regression. Furthermore, a nomogram incorporating the BMRGs score and other clinicopathological features was developed for accurate prediction of survival rate in patients with HCC.

**Results:**

A total of 121 differentially expressed BMRGs were screened from the TCGA HCC cohort. The functions of these BMRGs were significantly enriched in the extracellular matrix structure and signal transduction. The six-BMRGs risk score, comprising *CD151*, *CTSA*, *MMP1*, *ROBO3*, *ADAMTS5* and *MEP1A*, was established for the prediction of clinical prognosis, tumor microenvironment characteristics, and immunotherapy response in HCC. Kaplan-Meier analysis revealed that the BMRGs score-high group showed a significantly shorter overall survival than BMRGs score-low group. A nomogram showed that the BMRGs score could be used as a new effective clinical predictor and can be combined with other clinical variables to improve the prognosis of patients with HCC. Furthermore, the high BMRGs score subgroup exhibited an immunosuppressive state characterized by infiltration of macrophages and T-regulatory cells, elevated tumor immune dysfunction and exclusion (TIDE) score, as well as enhanced expression of immune checkpoints including PD-1, PD-L1, CTLA4, PD-L2, HAVCR2, and TIGIT. Finally, a multi-step analysis was conducted to identify two pivotal hub genes, *PKM* and *ITGA3*, in the high-scoring group of BMRGs, which exhibited significant associations with an unfavorable prognosis in HCC.

**Conclusion:**

Our study suggests that the BMRGs score can serve as a robust biomarker for predicting clinical outcomes and evaluating the tumor microenvironment in patients with HCC, thereby facilitating more effective clinical implementation of immunotherapy.

## Introduction

Hepatocellular carcinoma (HCC) is a common malignancy worldwide, with its incidence continuously increasing (1). In 2020, it was the third leading cause of cancer-related deaths (2). HCC has various histological types, making early detection and predicting postoperative recurrence vital for better patient outcomes (3). Despite treatments like chemotherapy, targeted therapy, immunotherapy, surgery, and liver transplantation, the prognosis remains poor with low five-year survival rates (4). Given HCC’s complex molecular characteristics and tumor microenvironment (TME), further investigation is needed. Understanding the molecular mechanisms of HCC and TME, identifying novel biomarkers for predicting clinical outcomes and serving as therapeutic targets are essential.

Basement membranes (BMs) are specialized components of the extracellular matrix, including laminins, type IV collagens, nidogens, proteoglycans, and growth factors. They provide structural support, determine tissue morphology, and act as diffusion barriers. BMs play crucial roles in tumor invasion and metastasis (5–8) and are targets for autoantibodies in immune diseases (9). Recent studies indicate that BM stiffness impacts metastatic development. For instance, the prognosis of breast cancer, kidney cancer, and melanoma is linked to the BM protein Netrin-4 (10). Changes or degradation in BM components are associated with poor tumor prognosis (11, 12). BMs significantly affect various types of tumors (13). However, research on the relationship between BM-related gene expression and clinicopathological features or prognosis in HCC is limited, necessitating further investigation.

The TME is a complex ecosystem surrounding the tumor (14), consisting of tumor cells and stromal components like extracellular matrix (ECM), BMs, vasculature, immune cells, and fibroblasts (15). During the early stages of tumor growth, dynamic interactions between cancer cells and TME components support cancer cell survival, local invasion, and metastasis (16). The cellular composition of HCC’s tumor-immune microenvironment (TIME) significantly affects tumor initiation, progression, and therapeutic response (17). Although the interaction between BMs and TME has been studied in various cancers, research on HCC remains limited.

In this study, we identified differentially expressed BM-related genes (BMRGs) between tumor and normal samples and investigated genes related to survival and prognosis. A prognostic risk model was developed using data from The Cancer Genome Atlas (TCGA), categorizing HCC patients into high-risk and low-risk groups based on median risk scores. This model was validated using the Gene Expression Omnibus (GEO) database to assess immune cell infiltration, gene mutations, drug sensitivity, and immunotherapy response between high-risk and low-risk patients. Additionally, a protein-protein interaction (PPI) network was constructed to identify the top 10 central genes. We explored the relationships between these central genes, immune cells, clinical traits, and survival prognosis. Our findings suggest that BMRGs could serve as therapeutic targets and prognostic indicators for HCC, and the risk scoring models may enable personalized treatment approaches.

## Materials and methods

### Data acquisition and compilation

This study utilized data from TCGA (https://portal.gdc.cancer.gov) (50 samples of healthy liver and 374 samples of HCC) and GEO (https://www.ncbi.nlm.nih.gov/geo/query/acc.cgi?acc=GSE14520) (242 HCC samples) databases to collect clinical information and transcription profiling data of patients with HCC. Gene IDs were converted into corresponding gene symbols using the human gene annotation file, with mean values used for multiple probes targeting the same gene ID. The TCGA dataset was used as the training set, while the GEO dataset served as the testing set. A total of 224 BMRGs previously identified in other studies were retrieved (**Supplementary Table 1**) (18), and their expression matrix was extracted for subsequent analysis after data matching, filtering, and correction.

### Enrichment analysis of the differentially expressed genes

The “Limma” R package was utilized for differential expression analysis of BMRGs in normal and cancer tissue samples, with statistical significance defined as FDR< 0.05 and LogFC< 0.585, resulting in the identification of 585 genes exhibiting significant differential expression. Subsequently, the differentially expressed genes (DEGs) underwent GO and KEGG pathway enrichment analysis using the “Clusterprofiler” R package to elucidate their biological characteristics and cellular functional pathways. Statistical significance was determined based on *P*-values and adjusted *P*-values< 0.05. Finally, visualization of the enrichment analysis results was performed using the “ggplot2” and “goplot” R packages. Gene mutation data were obtained from the TCGA database to calculate tumor mutation burden (TMB) in HCC patients, while exploration of DEG mutation frequency was conducted using the “Maftools” R package.

### Development and validation of a prognostic risk score model

First, the prognostic outcomes were integrated with the expression levels of DEGs in each sample. In the training set, univariate Cox regression analysis was employed to identify genes associated with prognosis among DEGs. Genes with *P-*values< 0.05 were selected for analyzing the association between gene mutation frequency and mutated genes in HCC samples from the training set using the “Maftools” R package. Subsequently, a Least Absolute Shrinkage Selection Operator (LASSO) Cox regression analysis was performed using the “glmnet” R package to further refine prognostic-related genes and develop a prognostic risk score model for predicting overall survival (OS) in HCC samples. The risk score for each sample was calculated using the following formula.


$$Risk\;score=\sum_{1}^{i}\left(\text{Coefi}*\text{ExpGenei}\right)$$

The “Coef” represents non-zero regression coefficients obtained through LASSO Cox regression analysis (**Supplementary Table 2**), while “ExpGene” denotes the expression value of a gene derived from a prognostic risk score model. All samples were stratified into high-risk and low-risk groups based on the median risk score, and Kaplan-Meier analysis was conducted using the log-rank test to compare overall survival differences between these two groups. Time-dependent receiver operating characteristic (ROC) curves were generated using the “Survival ROC” package in R to evaluate the predictive accuracy of prognostic risk scoring patterns. Finally, the validity and applicability of the prognostic risk scoring model were further assessed in an independent test set.

### Principal component analysis

To comprehensively investigate the distinct disparities between the high-risk and low-risk score groups, we successfully employed the “Limma” package in R to conduct principal component analysis (PCA) on the gene expression profiles before and after implementing the prognostic risk score model within our training dataset. Initially, PCA was performed on all DEGs associated with the basement membrane, followed by an examination of gene expression profiles derived from the prognostic risk score model using PCA. Ultimately, these PCA results were visually presented on a two-dimensional graph utilizing the “ggplot2” package.

### Association between risk score and clinical characteristics

The risk scores for each sample were integrated with the corresponding clinical information, encompassing gender, age, pathological stage, and TNM stage. The relationship between the risk score and clinical characteristics was investigated using the “limma” R package. Based on these clinical characteristics, the samples were stratified into two groups to facilitate comparison of differences in risk scores. Statistical significance was determined by considering *P*-values< 0.05.

### Reveal nomograms for prognosticating OS

The overall survival of individual patients with HCC was further investigated by developing nomograms using the “rms” package in R, incorporating age, gender, pathological stage, and prognostic risk score models. The predictive ability of these nomograms was evaluated through ROC curves and calibration plots.

### Characteristics of the high-risk group were compared with those of the low-risk group

Gene Set Enrichment Analysis (GSEA) was conducted on the “c2.cp.kegg.v7.4.symbols” gene sets obtained from the molecular signature database using the “org.Hs.eg.db” and “clusterProfiler” packages to assess differences in biological processes between high-risk and low-risk groups (https://www.gsea-msigdb.org/gsea/msigdb/). Additionally, single-sample gene set enrichment analysis (ssGSEA) was performed using the “GSVA” and “GSEABase” R packages to visualize infiltration fractions of 16 immune cells and activity levels of 13 immune-related pathways as ssGSEA scores. To predict the potential impact of immune checkpoint blockade, we also examined expression levels of PD-1, PD-L1, CTLA4, PD-L2, Havcr2, and TIGIT (19). Furthermore, we utilized the “PRRophetic” R package to predict semi-inhibitory concentrations of Sorafenib, Sunitinib, Erlotinib, and Gemcitabine in each sample as indicators for their effectiveness in inhibiting specific biological or biochemical functions (20). Lastly, we employed the Tumor Immune Dysfunction and Exclusion (TIDE) online database (http://TIDE.dfci.harvard.edu/) to forecast immunotherapy efficacy in both high-risk and low-risk populations; statistical significance was considered when *P*-value< 0.05 (21).

### Network of PPI

Firstly, the transcription profiling data of high-risk and low-risk scoring groups were compared using the ‘limma’ R package. Differentially expressed genes were identified based on a false discovery rate<0.05. The differentially expressed genes were then analyzed using the STRING online database (version: 11 5; https://cn.string-db.org/) to generate a PPI network with an interaction score >0.70 (high confidence). Subsequently, Cytoscape software (version: 3.9.1) was utilized for further processing and visualization of the PPI network data. The top 10 most central genes were screened using the cytoHubba plugin (version: 0.1). Next, all samples were stratified into high-expression and low-expression groups using the ‘cut off’ R package. Kaplan-Meier analysis was performed to assess survival differences between these two groups. Finally, we investigated immune cell infiltration patterns and clinical features associated with prognosis-related central genes.

### Statistical analysis

The Wilcoxon rank-sum test was employed to compare differences between the two groups. Kaplan-Meier analysis was utilized to evaluate survival disparities between high-risk and low-risk score groups. Multivariable Cox regression analysis was conducted to identify independent predictors of OS in HCC. ROC curves were plotted to assess the predictive validity of prognostic risk scoring models and nomograms. All statistical analyses were performed using R 4.0.2, with a significance level set at *P*< 0.05.

Additional materials and methods details are provided in the online **Supplementary Material**.

## Results

### Identification of differentially expressed BMRGs in HCC

**Figure 1:**
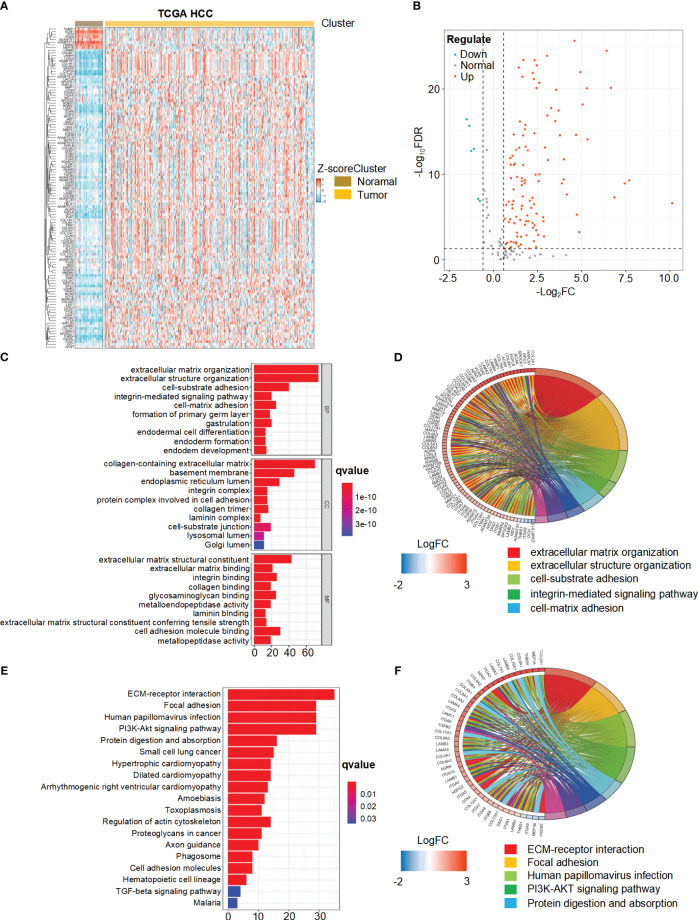
Differential expression and functional enrichment analysis of basement membrane-related genes (BMRGs) in hepatocellular carcinoma (HCC) patients from the TCGA Cohort. **(A)** Heatmap displaying the expression patterns of 121 BMRGs between tumor samples and normal samples in the TCGA-HCC cohort. **(B)** Volcano plot showing the differential expression of BMRGs between tumor and normal samples. **(C, D)** Go analysis of basement membranes (BMs)-related differential genes in HCC. **(E, F)** KEGG analysis of BMs-related differential genes in HCC.

We present the study’s flow chart in **Supplementary Figure 1**. A comprehensive analysis of the expression levels of 224 BMRGs in tumor and normal samples from the TCGA database, we identified 121 DEGs in the TCGA HCC cohort (*P*< 0.05, FDR< 0.585). Among these, 113 genes were upregulated, and 8 genes were downregulated in tumor samples. **Figures 1A** and **B** illustrate the heat map and volcano plot of DEGs between tumor and normal samples, respectively.

### Function and pathway enrichment analysis of DEGs

The biological functions of the differentially expressed BMRGs were further determined through GO annotation and KEGG pathway enrichment analyses. The identified BMRGs were primarily associated with extracellular matrix organization, extracellular structure organization, and cell-substrate adhesion in terms of biological processes (BP). In relation to cellular components (CC), the BMRGs were mainly enriched in collagen-containing extracellular matrix, basement membrane, and endoplasmic reticulum lumen. Regarding molecular functions (MF), the BMRGs predominantly exhibited roles as extracellular matrix structure constituents and cell adhesion molecule binders (**Figure 1C**). The top 80 significantly enriched genes and pathways are displayed in **Figure 1D**. KEGG pathway enrichment analysis revealed significant enrichments for ECM-receptor interaction, focal adhesion, human papillomavirus infection, and PI3K-Akt signaling pathway (**Figure 1E**). **Figure 1F** presents the top 45 significantly enriched genes and pathways. Collectively, these findings indicate a substantial correlation between BMRGs and extracellular matrix-associated functions and pathways in HCC progression.

### Development of the BMRGs

**Figure 2:**
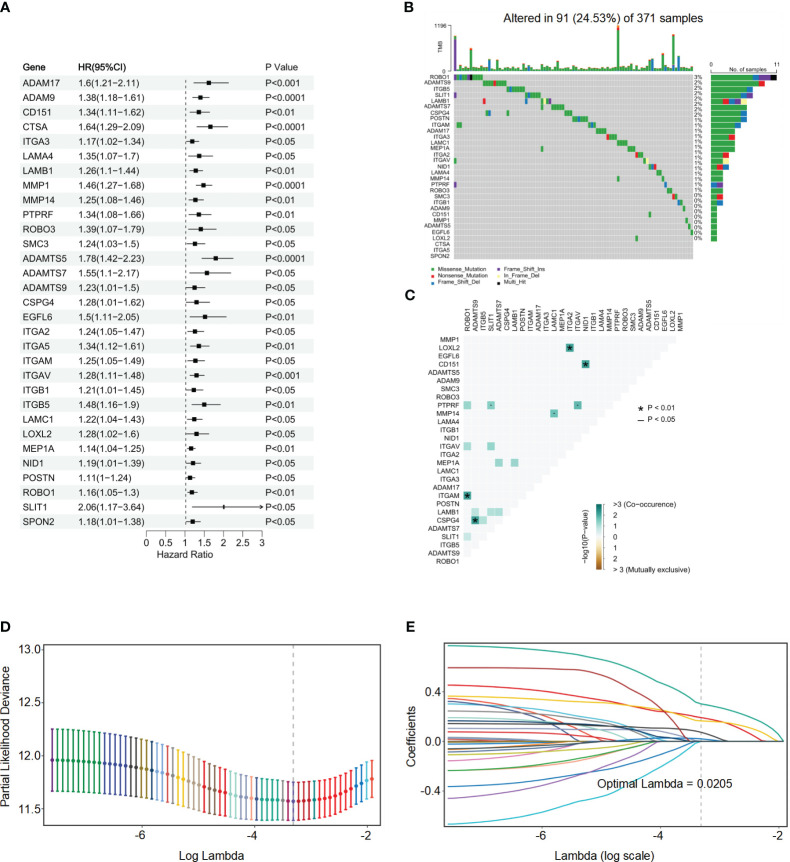
Univariate Cox regression analysis, mutation spectrum, and LASSO regression analysis of BMRGs in HCC. **(A)** Univariate Cox regression analysis of BMRGs in HCC patients from the TCGA cohort. **(B)** Mutation spectrum of the top 31 prognostic BMRGs in HCC patients. The waterfall plot shows the types and frequencies of mutations in these genes across 371 HCC samples. **(C)** Co-occurrence and mutual exclusivity analysis of mutations in the top 31 prognostic BMRGs. The heatmap shows significant correlations (*P*< 0.05) between gene mutations. **(D)** Partial likelihood deviance versus log (Lambda) for LASSO Cox regression analysis. **(E)** Coefficient profiles of the top 31 prognostic BMRGs from LASSO Cox regression analysis.

The TCGA-HCC cohort was utilized as the training set in this study, leading to the identification of 121 BMRGs. Univariate Cox analysis revealed 31 BMRGs significantly associated with OS (**Figure 2A**). Analysis of the somatic mutation spectrum in these prognostic genes demonstrated a mutation frequency of 24.53% in the HCC samples (n=371), with a total of 91 cases exhibiting mutations (**Figure 2B**). *ROBO1* exhibited the highest mutation frequency, followed by *ADAMTS9, ITGB5, SLIT1, Lamb1, ADAMTS7, CSPG4, POSTN, ITGAM, ADAM17, ITGA3, LAMC1, MEP1A, ITGA2, ITGAV, NID1, LAMA4, MMP14, PTPRF, ROBO3, SMC3* and *ITGB1*, while no mutations were observed in other genes. Further analysis revealed positive associations between *Robo1* and *ITGAM, ADAMTS9* and *CSPG4*, *ITGA2* and *LOXL2*, *NID1* and *CD151* (**Figure 2C**). These 31 genes were included in a LASSO Cox regression analysis based on the TCGA-HCC cohort. The analysis identified six genes, namely *CD151, CTSA, MMP1, ROBO3, ADAMTS5*, and *MEP1A*, which were used to construct the risk score model for BMRGs (**Figures 2D, E**). The risk score for each sample was calculated using the following formula: risk score = (0.00102 × expression of *CD151*) + (0. 192 × expression of *CTSA*) + (0. 165 × expression of *MMP1*) + (0.0739 × expression of *ROBO3*) + (0.302 × expression of *ADAMTS5*) + (0.0203 × expression of *MEP1A*).

### Prognostic significance of the BMRGs score

**Figure 3:**
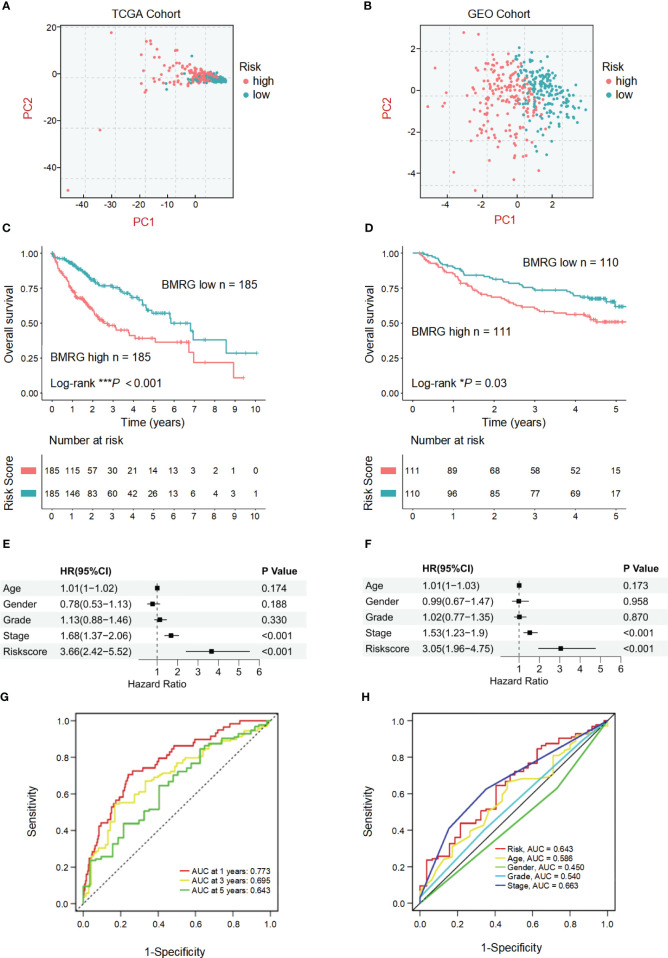
Validation and prognostic analysis of the BMRGs risk score model in HCC patients. **(A, B)** Principal component analysis (PCA) plots showing the distribution of high-risk and low-risk HCC patients in the TCGA cohort **(A)** and the GEO cohort **(B)**. **(C, D)** Kaplan-Meier survival curves displaying the overall survival (OS) of high-risk and low-risk HCC patients in the TCGA cohort **(C)** and the GEO cohort **(D)**. **(E, F)** Univariate and multivariate Cox regression analyses in the TCGA cohort **(E)** and the GEO cohort **(F)** showing that the risk score is an independent prognostic factor for OS in HCC patients (*P*< 0.001). **(G)** Area under the curve (AUC) value of HCC in 1, 3 and 5 years. **(H)** Receiver operating characteristic (ROC) curve of risk score and clinicopathological characteristics in HCC.

The established risk score model based on BMRGs was employed to effectively stratify the HCC cohorts into two distinct subgroups: high-risk and low-risk groups (**Figures 3A, B**). In this risk model, TCGA-HCC patients were classified into a high-risk group (n = 185) and a low-risk group (n = 185) using the median risk score as the cutoff, while GEO-HCC patients were divided into a high-risk group (n = 111) and a low-risk group (n = 110). Notably, in both the training and test sets, the low-risk group exhibited significantly improved clinical outcomes compared to the high-risk group (*P*-value< 0.05) (**Figures 3C, D**). The distribution of risk scores in relation to age, gender, pathological stage, and TNM stage of HCC was analyzed. Although there were no significant differences observed in the association between risk scores and age, gender, or M stage, higher risk scores were associated with elevated T, N, and pathological stages (**Supplementary Figure 2**). Univariate prognostic Cox analysis revealed that both pathological stage and risk score independently served as prognostic factors. This finding was further confirmed by multivariate Cox analysis (*P-*values< 0.05) (**Figures 3E, F**). The model’s reliability was assessed using ROC curves, yielding area under the curve (AUC) values of 0.773, 0.695, and 0.643 for years 1, 3, and 5, respectively (**Figure 3G**). Additionally, the risk model demonstrated an AUC of 0.643 (**Figure 3H**). These findings provide evidence supporting the reliability of the basement membrane risk model in predicting HCC progression.

### A nomograph for predicting survival

**Figure 4:**
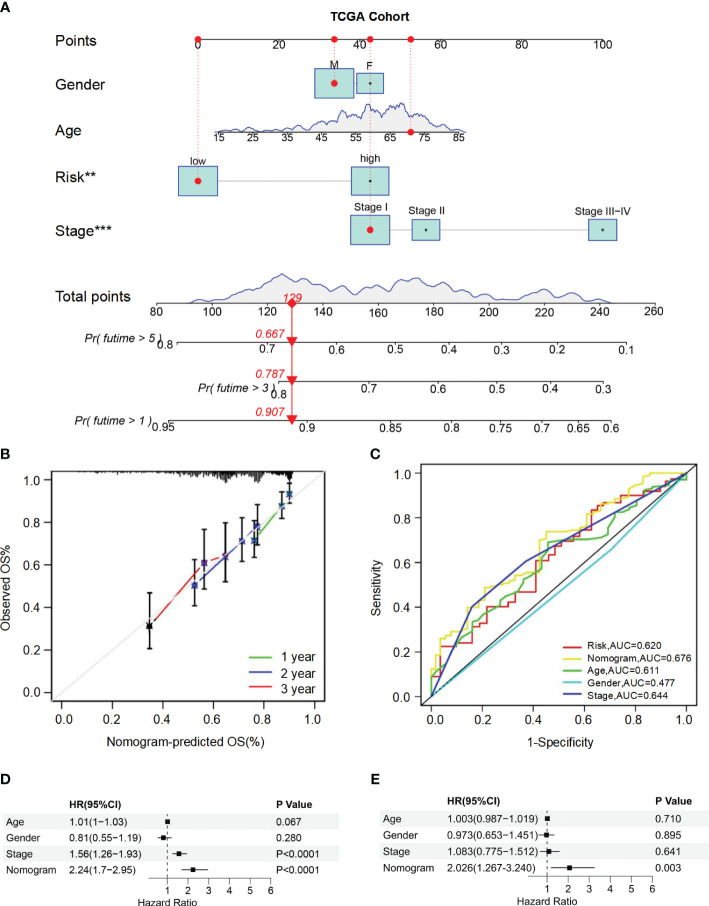
Construction and validation of the nomogram for predicting OS in HCC patients. **(A)** Nomogram for predicting 1-year, 3-year, and 5-year OS in HCC patients. **(B)** Calibration curves for the nomogram. The observed OS rates are plotted against the nomogram-predicted OS rates for 1-year, 2-year, and 3-year survival. **(C)** ROC curves for the nomogram, risk score, age, gender, and stage. **(D, E)** Univariate **(D)** and multivariate **(E)** Cox regression analyses of the nomogram and other clinical variables.

To accurately predict the probability of OS, we developed a nomogram that integrated the BMRGs score with other clinicopathological features, including gender, age, and pathological stage. The predictive performance of the nomogram for 1-year, 3-year, and 5-year OS in patients with HCC was assessed (**Figure 4A**). The calibration curve demonstrated close alignment with the ideal curve, indicating high predictive accuracy (**Figure 4B**). Furthermore, ROC curve analysis was conducted to validate the utility of the nomogram and calculate the AUC for risk assessment as well as for age, gender, and pathological stage prediction (**Figure 4C**). Notably, our nomogram exhibited the highest AUC value among all factors evaluated, indicating its superior prognostic accuracy. Univariate and multivariate Cox analyses performed on data from the training set confirmed that our nomogram served as an independent prognostic factor (*P*-value< 0.05) (**Figures 4D, E**). Collectively, these findings provide comprehensive validation of both the clinical applicability and predictive power associated with our BMRGs-based prognostic model.

### TME characteristics in different BMRGs subgroups

**Figure 5:**
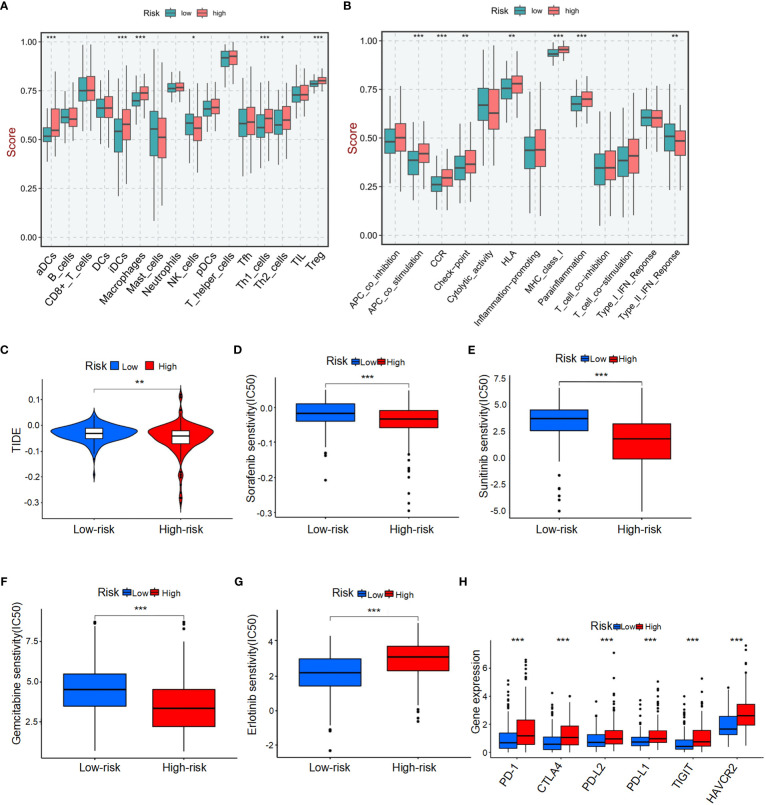
Tumor microenvironment (TME) characteristics and drug sensitivity in HCC patients stratified by BMRG risk score. **(A)** Immune cell infiltration in high-risk and low-risk patients. **(B)** Analysis of immune function in high-risk and low-risk patients. **(C)** Tumor immune dysfunction and exclusion (TIDE) scores for high-risk and low-risk patients. **(D-G)** Drug sensitivity analysis in high-risk and low-risk patients. **(H)** Analysis of differences in key immune checkpoints between high-risk and low-risk patients. The symbols *, **, and *** represent *P*-values of < 0.05, < 0.01, and < 0.001, respectively.

To comprehensively analyze TME, we utilized CIBERSORTx to calculate the infiltration degree of immune cell profiles. Our findings revealed a significant increase in the abundance of activated dendritic cells (aDCs), immature dendritic cells (iDCs), macrophages, T helper 1 (Th1) cells, T helper 2 (Th2) cells, and regulatory T cells (Tregs) in the BMRGs high-risk group. Conversely, the BMRGs low-risk group exhibited a significantly increased abundance of natural killer (NK) cells (**Figure 5A**). Immune function analysis demonstrated that APC co-stimulation, CCR signaling pathway activation, checkpoint regulation, HLA expression modulation, MHC class I antigen presentation enhancement and parainflammation-related functions were highly active in the high-risk group. In contrast, Type II IFN-Response immune-related functions were prominently activated in the low-risk group (**Figure 5B**). Furthermore, our results indicated that the high-risk group had lower Tumor Immune Dysfunction and Exclusion (TIDE) scores compared to the low-risk group (**Figure 5C**), suggesting enhanced effectiveness of immunotherapy within this subgroup. Risk score and drug sensitivity analysis revealed higher drug sensitivity to Sorafenib, Sunitinib, and Gemcitabine among patients classified into the high-risk group as opposed to those assigned to the low-risk category. However, Erlotinib displayed reduced drug sensitivity within the high-risk group (**Figures 5D-G**). Given its significance in checkpoint inhibitor immunotherapy approaches, we also investigated associations between risk scores and key immune checkpoints. Our findings indicated elevated levels of PD-1, PD-L1, CTLA4, PD-L2, HAVCR2, and TIGIT expression within individuals classified into the high-risk group (**Figure 5H**), thereby indicating an existence of immunosuppression within this subgroup. In conclusion, the quantification of BMRGs risk score represents a novel and reliable biomarker for evaluating prognosis associated with immunotherapy.

### PPI network of DEGs in high-risk and low-risk groups

**Figure 6:**
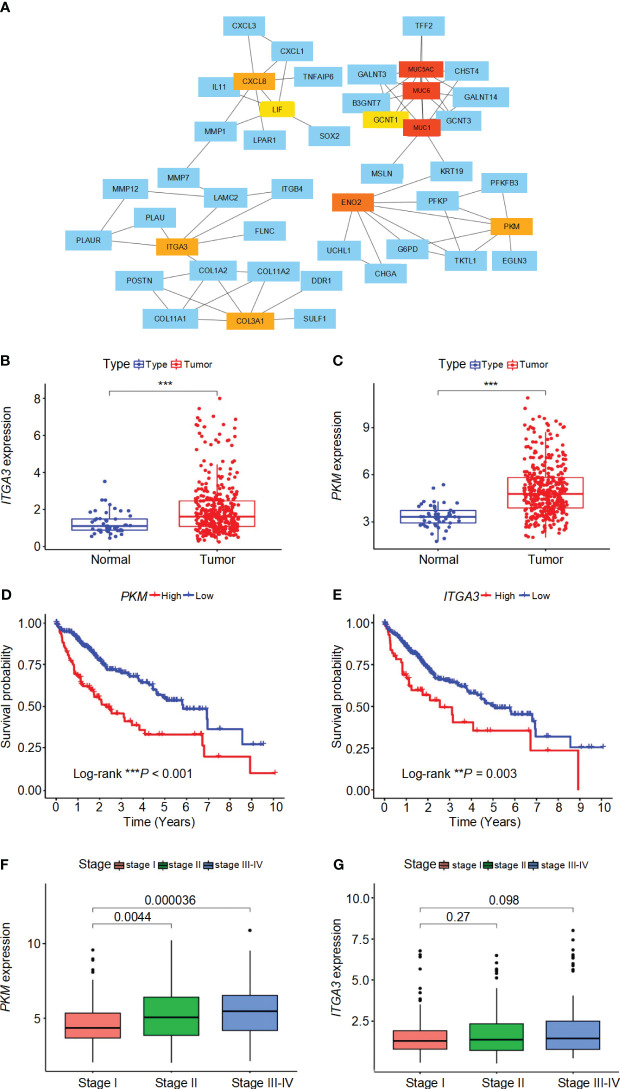
Interaction network, expression analysis, survival analysis, and stage-wise expression of *ITGA3* and *PKM* in HCC. **(A)** Protein-protein interaction (PPI) network of differentially expressed genes (DEGs) identified in high-risk and low-risk groups. The network was constructed using the STRING database and visualized with Cytoscape software. **(B)** Comparison of *ITGA3* expression levels between normal and tumor samples. **(C)** Comparison of *PKM* expression levels between normal and tumor samples. **(D)** Survival curves for patients with high and low *PKM* expression. **(E)** Survival curves for patients with high and low *ITGA3* expression. **(F)** Comparison of *PKM* expression levels across different tumor stages. **(G)** Comparison of *ITGA3* expression levels across different tumor stages. The symbols *** represent *P*-values of < 0.001.

The interactions between DEGs in high-risk and low-risk groups were analyzed using the STRING online database, revealing a complex PPI network (**Supplementary Figure 3A**). The PPI network data were further processed and visualized using Cytoscape software, highlighting the intricate interactions among DEGs (**Supplementary Figure 3B**). Hub genes were identified using the cytoHubba plugin in Cytoscape, resulting in the selection of ten key genes within the network, namely *MUC1*, *MUC6*, *MUC5AC*, *ENO2*, *PKM*, *CXCL8*, *ITGA3*, *COL3A1*, *GCNT1*, and *LIF* (**Figure 6A**). These hub genes were sequenced using the degree method, as detailed in **Supplementary Table 2**. Differential analysis revealed that *PKM* and *ITGA3* are significantly overexpressed in HCC patients compared to normal samples (*P*< 0.05) (**Figures 6B, C**). Moreover, both *PKM* and *ITGA3* showed a significant association with survival outcomes, where higher gene expression correlated with worse prognosis (*P*< 0.05) (**Figures 6D, E**). Furthermore, *PKM* expression was found to increase with tumor stage progression, indicating its potential involvement in the advancement of HCC (**Figure 6F**). In contrast, *ITGA3* expression did not show a significant correlation with tumor stage, although a decreasing trend in advanced stages was observed (**Figure 6G**). These results suggest that *PKM* and *ITGA3* could serve as potential biomarkers and therapeutic targets for HCC.

### *PKM2*/*ITGA3* are significant upregulated in HCC samples and predicts poor prognosis

**Figure 7:**
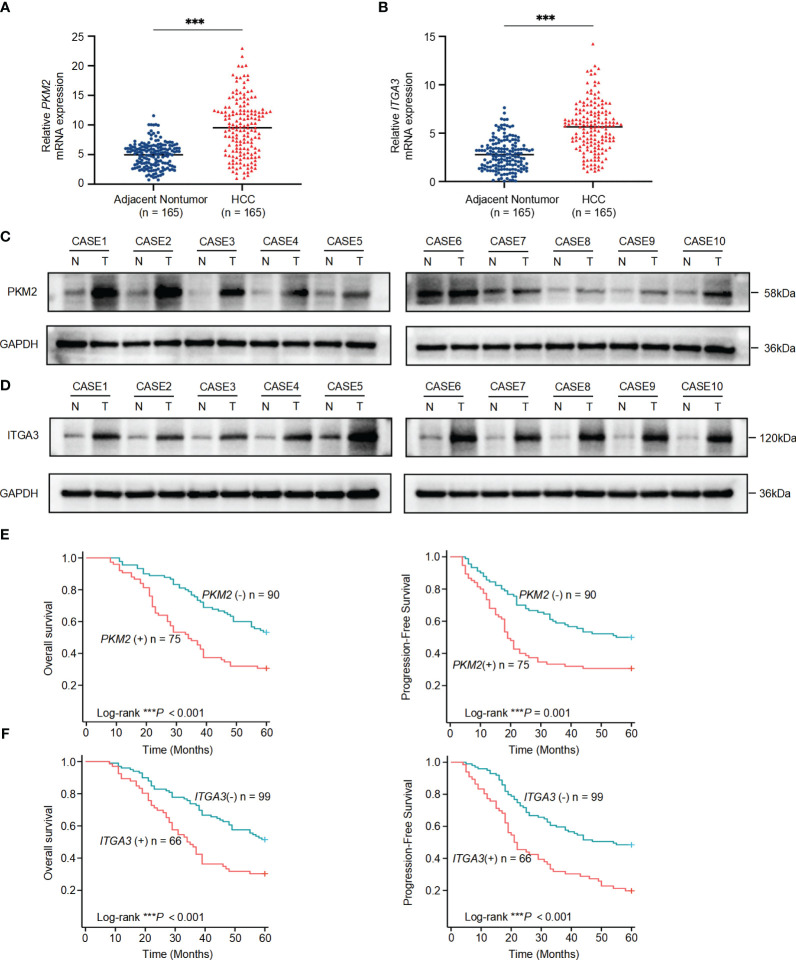
Significant upregulation of *PKM2* and *ITGA3* in HCC tissues and their prognostic implications. **(A)** Relative mRNA expression levels of *PKM2* in 165 paired HCC and adjacent nontumor specimens, measured by quantitative RT-PCR. **(B)** Relative mRNA expression levels of *ITGA3* in the same 165 paired HCC and adjacent nontumor specimens, measured by quantitative RT-PCR. **(C, D)** Western blot analysis of PKM2 **(C)** and ITGA3 **(D)** protein levels in HCC and adjacent nontumor specimens. **(E)** Kaplan-Meier survival curves showing overall survival (left) and progression-free survival (right) of HCC patients based on *PKM2* expression levels. **(F)** Kaplan-Meier survival curves showing overall survival (left) and progression-free survival (right) of HCC patients based on *ITGA3* expression levels. The symbols *** represent *P*-values of < 0.001.

To further validate the association of *PKM2* and *ITGA3* with HCC at the clinical level, we first examined the mRNA levels of *PKM2* and *ITGA3* in 165 paired HCC and corresponding adjacent nontumor specimens by quantitative RT-PCR (RT-qPCR). *PKM2* and *ITGA3* mRNA levels were significantly elevated in HCC specimens compared to adjacent nontumor specimens (**Figures 7A, B**).

We then assessed the protein levels of PKM2 and ITGA3 in paired HCC specimens. Western blot assay showed that PKM2 and ITGA3 were significantly elevated in HCC specimens compared to adjacent nontumor specimens (**Figures 7C, D**).

To explore the clinical significance of *PKM2* and *ITGA3*, HCC patients were categorized into two groups based on RT-qPCR, negative or positive of *PKM2*/*ITGA3*. Notably, Kaplan-Meier analysis revealed that higher *PKM2* or *ITGA3* levels were associated with worse OS and worse Progression-Free Survival (PFS) in HCC patients (**Figures 7E, F**). Clinicopathological characterization indicated that *PKM2* or *ITGA3* overexpression was associated with age, serum AFP, TNM stage, BCLC stage, tumor number, tumor size, and microvascular invasion (**Table 1**). Multivariate analysis demonstrated that *PKM2* and *ITGA3* are significant and independent predictors of OS and PFS in HCC patients (**Supplementary Tables 3**, **4**). In conclusion, our data suggest that regulation of *PKM2* and *ITGA3* may predict poor prognosis in HCC patients.

**Table 1:** Correlation between PKM2/ITGA3 and clinicopathological characteristics of HCC in human HCC tissues from independent cohorts.

Variables		PKM2 expression			ITGA3 expression		
		Negative (n = 90)	Positive (n = 75)	*P* Value	Negative (n = 99)	Positive (n = 66)	*P* Value
Age	≤60 years	49	26	0.011	55	20	0.001
	>60 years	41	49		44	46	
Sex	Female	28	21	0.663	28	21	0.626
	Male	62	54		71	45	
HBV	No	26	15	0.188	25	16	0.883
	Yes	64	60		74	50	
Serum AFP	≤400ng/ml	68	44	0.021	74	38	0.021
	>400ng/ml	22	31		25	28	
^a^TNM stage	I-II	84	55	<0.001	90	49	0.004
	III	6	20		9	17	
^b^BCLC stage	0-A	78	50	0.002	85	43	0.002
	B~C	12	25		14	23	
Tumor number	Single	73	46	0.005	81	38	0.001
	Multiple	17	29		18	28	
Tumor size	≤5cm	56	33	0.019	64	25	0.001
	>5cm	34	42		35	41	
Microvascular invasion	Absent	65	38	0.004	68	35	0.042
	Present	25	37		31	31	
Cirrhosis	Absent	41	19	0.007	43	17	0.021
	Present	49	56		56	49	

HBV, hepatitis B virus; AFP, alpha-fetoprotein; TNM, tumor–node–metastasis; BCLC, Barcelona Clinic Liver Cancer; HR, hazard ratio; CI, confidence interval.

a American Joint Committee on Cancer 8th edition staging for hepatocellular carcinoma.

b Barcelona Clinic Liver Cancer systems, 2022.

### GSEA of the BMRGs

To investigate the biological functions associated with BMRGs, we employed GSEA to analyze the TCGA cohort. We identified 37 pathways that were significantly enriched in the high-risk group and 8 pathways in the low-risk group (*P*< 0.05). Notably, the top five enrichment pathways in the high-risk group included cell adhesion molecules (CAMs), cytokine-cytokine receptor interaction, ECM-receptor interaction, focal adhesion, and neuroactive ligand-receptor interaction (**Supplementary Figure 4A**). Conversely, the top five enrichment pathways in the low-risk group comprised beta-alanine metabolism, fatty acid metabolism, linoleic acid metabolism, primary bile acid biosynthesis and retinol metabolism (**Supplementary Figure 4B**). These findings underscore the distinct biological processes that are activated in high-risk and low-risk groups, providing insights into the molecular mechanisms underlying the progression and treatment response of HCC.

## Discussion

Despite significant advancements in early detection, targeted therapy, and immunotherapy for HCC over the past decades, OS in HCC patients remain low. Therefore, there is an urgent need to comprehensively understand the pathogenesis and mechanisms underlying HCC development and identify potential clinical therapeutic targets. BMs play a pivotal role in regulating cell polarity, differentiation, migration, and survival processes (22, 23). BM proteins are also the targets of autoantibodies in immune diseases, and defective expression of BM proteins is a key pathogenic aspect of cancer, diabetes, and fibrosis (24, 25). Previous studies have demonstrated a significant association between BM components and cancer progression, thus they can be considered as potential targets for inhibiting tumor growth (10–12). Although numerous studies have utilized molecular markers based on genes to predict prognosis in HCC patients (26), no studies have systematically utilized BMRGs to predict the prognosis of HCC patients. Exploring the role of different BMRGs in HCC is helpful to understand the role of BMs in the occurrence and development of HCC and to guide effective treatment strategies.

In this study, we employed univariate Cox regression analysis and LASSO Cox regression analysis to establish a prognostic risk score model based on the differential expression of 121 BMRGs in tumor and normal liver tissue samples from the TCGA and GEO cohorts. This model was utilized to predict OS in patients with HCC, aiming to gain deeper insights into the role of these genes in HCC pathogenesis. Significant differences were observed in the survival outcomes between high-risk and low-risk score groups among HCC patients, which were further validated using an independent dataset. These findings suggest that the prognostic risk score model holds potential for identifying patients with poor survival, while also serving as an independent prognostic factor according to multivariate analysis. Furthermore, we constructed a nomogram that effectively evaluated clinical survival for individual patients. Subsequently, functional enrichment analysis revealed a correlation between differential expression of BMRGs and the occurrence and progression of HCC.

Immunotherapy aims to harness the intrinsic immune molecules within TME for cancer prevention. In our analysis of immune function, we observed distinct TME among different risk groups of HCC. Specifically, in the high-risk group, most immune cell populations exhibited elevated levels except for NK cells. Furthermore, the activity of various other immune pathways was significantly higher compared to the Type II IFN Response pathway. Consequently, we hypothesize that the diminished presence of NK cells in high-risk populations may contribute to their survival disadvantage. This highlights a promising avenue for future research, stimulating NK cell-mediated immune responses and reinforcing immunotherapy alongside other treatment strategies to effectively eliminate tumor cells. Notably, activation of type II interferon (IFN) response in the low-risk group underscores its close association with HCC progression. Interferons are classified into three types, type I (IFN-α and IFN-β), type II (IFN-γ), and Type III (27). IFN-α represents a crucial therapeutic approach for patients with liver cancer (28). The activation of interferon-stimulated gene (ISG) transcription is initiated by the binding of IFN-α to its receptor and subsequent mediation of signal transduction. These genes determine the biological outcomes associated with STAT1 signal transduction, including immune function modulation, cell proliferation inhibition, and apoptosis induction (29).Recent research has demonstrated that IFN-α can effectively impede HCC growth and induce apoptosis (30). IFN-γ is primarily released by antigen-recognized and activated T cells (31), which triggers *B7-H1* gene expression in lung cancer cells, cholangiocarcinoma cells, head and neck cancers, as well as HCC through the JAK/STAT1 pathway (31–34). NK cell-secreted IFN-γ plays a significant role in maintaining dormancy to prevent liver metastasis while also preserving dormancy itself (35), aligning with the survival advantage observed in low-risk groups.

Risk score and drug sensitivity analysis revealed that patients with a high-risk score exhibited sensitivity to three commonly used drugs for HCC, namely Sorafenib, Sunitinib, and Gemcitabine. Conversely, patients with a low-risk score demonstrated increased sensitivity to Erlotinib. These findings present a novel approach for guiding clinical treatment of liver cancer. Our study identified an association between different risk scores and the expression of immune checkpoint molecules in HCC, including PD-1, PD-L1, CTLA4, PD-L2, HAVCR2, and TIGIT. Notably, PD-1 may play a crucial role in promoting cancer development. Combining PD-1 blockade with mTOR pathway targeting could potentially enhance the antitumor efficacy against cancer (36, 37). Moreover, elevated expression of PD-L1 in HCC leads to exhaustion of follicular helper T-cells and impairs cytokine expression as well as B-cell help, functionally contributing to tumor progression towards advanced stages (38). Consequently, patients with high-risk scored HCC might derive benefits from immune checkpoint inhibitor therapy.

PPI network was constructed based on the DEGs between high-risk and low-risk groups, and subsequently, 10 hub genes were identified. Significantly, *PKM* and *ITGA3* exhibited statistical significance (*P*< 0.05) in both differential analysis and survival analysis. These two genes are highly expressed in HCC and belong to the high-risk gene category. Moreover, higher expression levels of these genes correlate with worse prognosis. Specifically, pyruvate kinase M2 isoform (PKM2), a crucial enzyme involved in glycolysis, facilitates glucose conversion into lactic acid within cancer cells under aerobic conditions (39, 40).. Additionally, phosphorylation of PKM2 along with STAT3 inhibition has been shown to impede lung cancer cell proliferation effectively (41). The PKM2 inhibitor can moderately attenuate the proliferation of tumor cells (42, 43). In a HIF-1α-dependent manner, the key glycosylase PKM2, induced by liver cancer cell-derived fibrin 1, simultaneously regulates the antitumor properties of glycosylated macrophages and inflammation-mediated PD-L1 expression. Importantly, while an increase in PKM2-regulated glycolytic macrophages predicts a poor prognosis for patients, blocking PD-L1 on these cells abolishes PD-L1 dominated immunosuppression and unleashes intrinsic antitumor properties (44). This finding is consistent with our current results. *ITGA3*, also known as integrin α3, belongs to the integrin family and interacts with numerous ECM proteins. It mediates cell-cell adhesion and cell-matrix adhesion while bridging external and internal cellular structures (45). Although *ITGA3* is widely expressed in normal organisms, under the influence of tumor gene induction, changes in chromatin structure along with high expression of growth factors and their receptors disrupt its expression pattern leading to tumorigenesis. Notably, *ITGA3* has been identified as a negative prognostic factor for pancreatic cancer (46), head and neck cancer (47), and tongue squamous cell carcinoma (48). These two central gene activation states exhibit an increasing infiltration of active immune cells, suggesting that immunotherapy may potentially modify survival outcomes in patients with a poor prognosis.

Our clinical sample study further validated these findings. The *PKM* gene produces two protein isoforms through alternative splicing, the M2 isoform *(PKM2*) is expressed at higher levels than the M1 isoform (*PKM1*), and *PKM2* is the predominant isoform in all human cancer cell lines (49). Therefore, we chose *PKM2* for further validation. *PKM2* and *ITGA3* are significant upregulated in HCC tissues and are closely associated with poor clinicopathological characteristics and prognosis. Future research should aim to validate and expand upon the findings of this study, focusing on the mechanistic roles of BMs, developing targeted therapies, optimizing combination treatments, and integrating multi-omics data. Such efforts will enhance our understanding of HCC and improve therapeutic strategies, ultimately leading to better patient outcomes.

In conclusion, we have developed a risk model for BMRGs in HCC patients. Our study suggests that the BMRGs score can serve as a robust biomarker for predicting clinical outcomes and evaluating the tumor microenvironment in patients with HCC. This may help clinicians to better assess the prognosis of patients, formulate personalized treatment strategies, and ultimately improve the therapeutic outcomes and survival rates of HCC patients.

## Data availability statement

The raw data supporting the conclusions of this article will be made available by the authors, without undue reservation.

## Author contributions

BL: Methodology, Validation, Writing – original draft, Writing – review & editing. YC: Data curation, Software, Writing – original draft, Writing – review & editing. PZ: Investigation, Writing – review & editing. YX: Writing – review & editing. HY: Writing – review & editing. DL: Conceptualization, Funding acquisition, Methodology, Writing – review & editing. XD: Conceptualization, Funding acquisition, Methodology, Writing – review & editing.
