# Supplementary material for: A novel basement membrane-related gene signature predicts prognosis and immunotherapy response in hepatocellular carcinoma
Source: Front Oncol. 2024 Jul 12;14:1388016. doi: 10.3389/fonc.2024.1388016 (PMC11272612; doi:10.3389/fonc.2024.1388016)
Supplement: Supplementary file 1 [file DataSheet_1.docx]

**Supplementary Materials**

**Supplementary Methods**

**Patient samples and ethics statement**

# This study was approved by the Ethics Committee of Henan Provincial People's Hospital and was conducted in accordance with the ethical standards of the Declaration of Helsinki of the World Medical Association. The validation cohort data included 165 HCC patients who underwent curative resection at Henan Provincial People's Hospital between 2011 and 2018.

**Quantitative reverse-transcription (RT-q) PCR**

Total RNA was extracted from tissues using TRIzol Reagent (TaKaRa) according to the manufacturer's protocol. The extracted RNA was reverse-transcribed into cDNA using the PrimeScript RT Reagent Kit (TaKaRa) following the manufacturer's instructions. Quantitative PCR (qPCR) was performed using SYBR Premix ExTaq (TaKaRa) on an ABI StepOne Real-Time PCR System (Applied Biosystems, Carlsbad, CA, USA).The qPCR reactions were set up in a total volume of 20 µL, containing 10 µL of SYBR Premix ExTaq, 0.4 µL of forward primer (10 µM), 0.4 µL of reverse primer (10 µM), an appropriate amount of cDNA template, and nuclease-free water to adjust the final volume. The thermal cycling conditions were as follows: initial denaturation at 95°C for 30 seconds, followed by 40 cycles of 95°C for 5 seconds and 60°C for 30 seconds. Relative gene expression levels were quantified using the 2^–ΔΔCt method, with normalization to an appropriate endogenous control gene. Fold changes in expression levels between tissues were calculated to determine the relative differences.

# Western Blot Analysis

# Protein from lysate cells for each group was prepared in RIPA buffer with the addition of protease inhibitor (Sigma-Aldrich), followed by placement on ice for 30 min. After the centrifugation at 13,000 rpm at 4 °C for 10 min, the supernatants were denatured and loaded on 10% SDS-PAGE gel. Proteins were then transferred in an ice bath to polyvinylidene difluoride membrane (Millipore). After blocking with TBST buffer containing 5% milk for 1 h, the membrane was incubated with primary antibodies overnight at 4 °C, followed by secondary antibody 2h at room temperature. Western blotting of GAPDH (Proteintech) on the same membrane was used as a loading control. The antibodies were as follows: PKM2 (Proteintech) and ITGA3 (Proteintech).

**Supplementary Tables**

**Supplementary Table 1.** 224 basement membrane-related genes

| 224 basement membrane-related genes | | | | |
| --- | --- | --- | --- | --- |
| ACAN | DDR2 | P3H2 | EGFLAM | MMRN2 |
| ADAM10 | ECM1 | PTPRF | EVA1A | NELL1 |
| ADAM17 | EFEMP1 | PXDN | EVA1B | NELL2 |
| ADAM9 | EFEMP2 | ROBO2 | EVA1C | NID1 |
| ADAMTS10 | FBLN1 | ROBO3 | FBLN2 | NID2 |
| ADAMTS13 | FBLN5 | ROBO4 | FBN3 | NPNT |
| ADAMTS17 | FBN1 | RPSA | FMOD | NTN4 |
| ADAMTS18 | FBN2 | SERPINF1 | FREM3 | OGN |
| ADAMTS2 | FGF9 | SMC3 | GPC1 | OPTC |
| ADAMTS3 | FN1 | SMOC1 | GPC2 | PAPLN |
| AGRN | FRAS1 | SMOC2 | GPC5 | PHF13 |
| AMELX | FREM1 | SPARC | HAPLN1 | PODN |
| AMTN | FREM2 | TENM3 | HAPLN2 | POSTN |
| ANG | GPC3 | TENM4 | HMCN2 | PTN |
| BGN | GPC4 | TGFB1 | ISLR | PXDNL |
| CD151 | GPC6 | TGFB2 | ITGA1 | RECK |
| CERT1 | HMCN1 | TGFBI | ITGA10 | ROBO1 |
| COL12A1 | HSPG2 | TIMP3 | ITGA2 | SDC1 |
| COL13A1 | ITGA2B | TLL1 | ITGA4 | SDC4 |
| COL17A1 | ITGA3 | TNC | ITGA5 | SEMA3B |
| COL18A1 | ITGA6 | USH2A | ITGA9 | SLIT1 |
| COL2A1 | ITGA7 | VCAN | ITGAM | SLIT2 |
| COL4A1 | ITGA8 | ACHE | ITGAV | SLIT3 |
| COL4A2 | ITGB2 | ADAMTS1 | ITGAX | SPARCL1 |
| COL4A3 | ITGB3 | ADAMTS14 | ITGB1 | SPOCK1 |
| COL4A4 | ITGB4 | ADAMTS15 | ITGB5 | SPOCK2 |
| COL4A5 | ITGB6 | ADAMTS16 | ITGB7 | SPOCK3 |
| COL4A6 | LAMA1 | ADAMTS19 | ITGB8 | SPON1 |
| COL5A1 | LAMA2 | ADAMTS20 | LAD1 | SPON2 |
| COL6A1 | LAMA3 | ADAMTS4 | LAMA5 | TENM1 |
| COL6A2 | LAMA4 | ADAMTS5 | LAMB4 | TENM2 |
| COL6A3 | LAMB1 | ADAMTS6 | LAMC1 | THBS1 |
| COL7A1 | LAMB2 | ADAMTS7 | LOXL2 | THBS2 |
| COL8A2 | LAMB3 | ADAMTS8 | LOXL4 | THBS4 |
| COL9A1 | LAMC2 | ADAMTS9 | LUM | TIMP1 |
| COL9A2 | LAMC3 | BCAN | MATN1 | TIMP2 |
| COL9A3 | LOXL1 | CCDC80 | MATN2 | TINAG |
| COLQ | MMP1 | CD44 | MATN4 | TINAGL1 |
| CST3 | MMP14 | COL14A1 | MEGF6 | UNC5A |
| CTSA | MMP2 | COL15A1 | MEGF9 | UNC5B |
| CTSB | MMP21 | COL28A1 | MEP1A | UNC5C |
| CTSD | MPZL2 | COL8A1 | MEP1B | UNC5D |
| DAG1 | MUSK | CSPG4 | MMP17 | VTN |
| DCC | NTN1 | DDR1 | MMP26 | VWA1 |
| DCN | P3H1 | EGFL6 | MMP7 |  |

**Supplementary Table 2.** Non-zero regression coefficients calculated using the LASSO Cox regression analysis

| Gene | Coef |
| --- | --- |
| CD151 | 0.001022 |
| CTSA | 0.191942 |
| MMP1 | 0.165404 |
| ROBO3 | 0.073859 |
| ADAMTS5 | 0.301797 |
| MEP1A | 0.020292 |

**Supplementary Table 3.** Univariate and multivariate analyses of factors associated with survival and progression-free survival in Cohort HCCs (n = 165)

|  | Overall Survival | |  | Progression-Free Survival | |
| --- | --- | --- | --- | --- | --- |
| Clinical Variables | HR (95%CI) | *P* value |  | HR (95%CI) | *P* value |
| **Univariate Analysis** |  |  |  |  |  |
| Age (≤60 versus >60 years) | 0.636 (0.420-0.965) | 0.033 |  | 0.658 (0.444-0.974) | 0.037 |
| Sex (female versus male) | 0.930 (0.592-1.462) | 0.753 |  | 0.901 (0.584-1.390) | 0.636 |
| HBV infection (absent versus presents) | 0.863 (0.531-1.402) | 0.551 |  | 0.801 (0.500-1.283) | 0.356 |
| Serum AFP (≤400 versus >400 ng/ml) | 0.484 (0.321-0.730) | 0.001 |  | 0.485 (0.327-0.718) | <0.001 |
| ^a^TNM stage ((I-II versus III) | 0.548 (0.331-0.909) | 0.020 |  | 0.538 (0.332-0.870) | 0.012 |
| ^b^BCLC stage (0-A versus B~C) | 0.586 (0.376-0.913) | 0.018 |  | 0.502 (0.330-0.765) | 0.001 |
| Tumor number (single versus multiple) | 0.629 (0.410-0.964) | 0.033 |  | 0.640 (0.425-0.965) | 0.033 |
| Tumor size (≤5 versus >5 cm) | 0.662 (0.441-0.992) | 0.046 |  | 0.656 (0.447-0.965) | 0.032 |
| Microvascular invasion (absent versus present) | 0.582 (0.387-0.875) | 0.009 |  | 0.604 (0.410-0.892) | 0.011 |
| Cirrhosis (absent versus present) | 0.566 (0.360-0.891) | 0.014 |  | 0.539 (0.349-0.833) | 0.005 |
| PKM2 (negative versus positive) | 0.476 (0.316-0.717) | **<0.001** |  | 0.420 (0.284-0.622) | **<0.001** |
| ^c^**Multivariate Analysis** |  |  |  |  |  |
| Serum AFP (≤400 versus >400 ng/ml) | 0.512 (0.336-0.780) | 0.002 |  | 0.522 (0.349-0.781) | 0.002 |
| Microvascular invasion (absent versus present) | 0.622 (0.408-0.948) | 0.027 |  | 0.652 (0.437-0.973) | 0.036 |
| PKM2 (negative versus positive) | 0.588 (0.384-0.901) | **0.015** |  | 0.503 (0.336-0.753) | **0.001** |

| Abbreviations: HBV, hepatitis B virus; AFP, alpha-fetoprotein; TNM, tumor–node–metastasis; BCLC, Barcelona Clinic Liver Cancer; HR, hazard ratio; CI, confidence interval. |
| --- |
| ^a^American Joint Committee on Cancer 8th edition staging for hepatocellular carcinoma. |
| ^b^Barcelona Clinic Liver Cancer systems, 2022 |
| ^c^Cox proportional hazards regression analysis adjusting using forward:LR selection. |

**Supplementary Table 4.** Univariate and multivariate analyses of factors associated with survival and progression-free survival in Cohort HCCs (n = 165)

|  | Overall Survival | |  | Progression-Free Survival | |
| --- | --- | --- | --- | --- | --- |
| Clinical Variables | HR (95%CI) | *P* value |  | HR (95%CI) | *P* value |
| **Univariate Analysis** |  |  |  |  |  |
| Age (≤60 versus >60 years) | 0.636 (0.420-0.965) | 0.033 |  | 0.658 (0.444-0.974) | 0.037 |
| Sex (female versus male) | 0.930 (0.592-1.462) | 0.753 |  | 0.901 (0.584-1.390) | 0.636 |
| HBV infection (absent versus presents) | 0.863 (0.531-1.402) | 0.551 |  | 0.801 (0.500-1.283) | 0.356 |
| Serum AFP (≤400 versus >400 ng/ml) | 0.484 (0.321-0.730) | 0.001 |  | 0.485 (0.327-0.718) | <0.001 |
| ^a^TNM stage ((I-II versus III) | 0.548 (0.331-0.909) | 0.020 |  | 0.538 (0.332-0.870) | 0.012 |
| ^b^BCLC stage (0-A versus B~C) | 0.586 (0.376-0.913) | 0.018 |  | 0.502 (0.330-0.765) | 0.001 |
| Tumor number (single versus multiple) | 0.629 (0.410-0.964) | 0.033 |  | 0.640 (0.425-0.965) | 0.033 |
| Tumor size (≤5 versus >5 cm) | 0.662 (0.441-0.992) | 0.046 |  | 0.656 (0.447-0.965) | 0.032 |
| Microvascular invasion (absent versus present) | 0.582 (0.387-0.875) | 0.009 |  | 0.604 (0.410-0.892) | 0.011 |
| Cirrhosis (absent versus present) | 0.566 (0.360-0.891) | 0.014 |  | 0.539 (0.349-0.833) | 0.005 |
| ITGA3 (negative versus positive) | 0.513 (0.342-0.771) | **0.001** |  | 0.444 (0.302-0.654) | **<0.001** |
| ^c^**Multivariate Analysis** |  |  |  |  |  |
| Serum AFP (≤400 versus >400 ng/ml) | 0.514 (0.335-0.788) | 0.002 |  | 0.574 (0.377-0.872) | 0.009 |
| Microvascular invasion (absent versus present) | 0.587 (0.388-0.890) | 0.012 |  | 0.594 (0.398-0.887) | 0.011 |
| ITGA3 (negative versus positive) | 0.648 (0.425-0.990) | **0.045** |  | 0.598 (0.396-0.904) | **0.015** |

| Abbreviations: HBV, hepatitis B virus; AFP, alpha-fetoprotein; TNM, tumor–node–metastasis; BCLC, Barcelona Clinic Liver Cancer; HR, hazard ratio; CI, confidence interval. |
| --- |
| ^a^American Joint Committee on Cancer 8th edition staging for hepatocellular carcinoma. |
| ^b^Barcelona Clinic Liver Cancer systems, 2022 |
| ^c^Cox proportional hazards regression analysis adjusting using forward:LR selection. |

**Supplementary Figures**


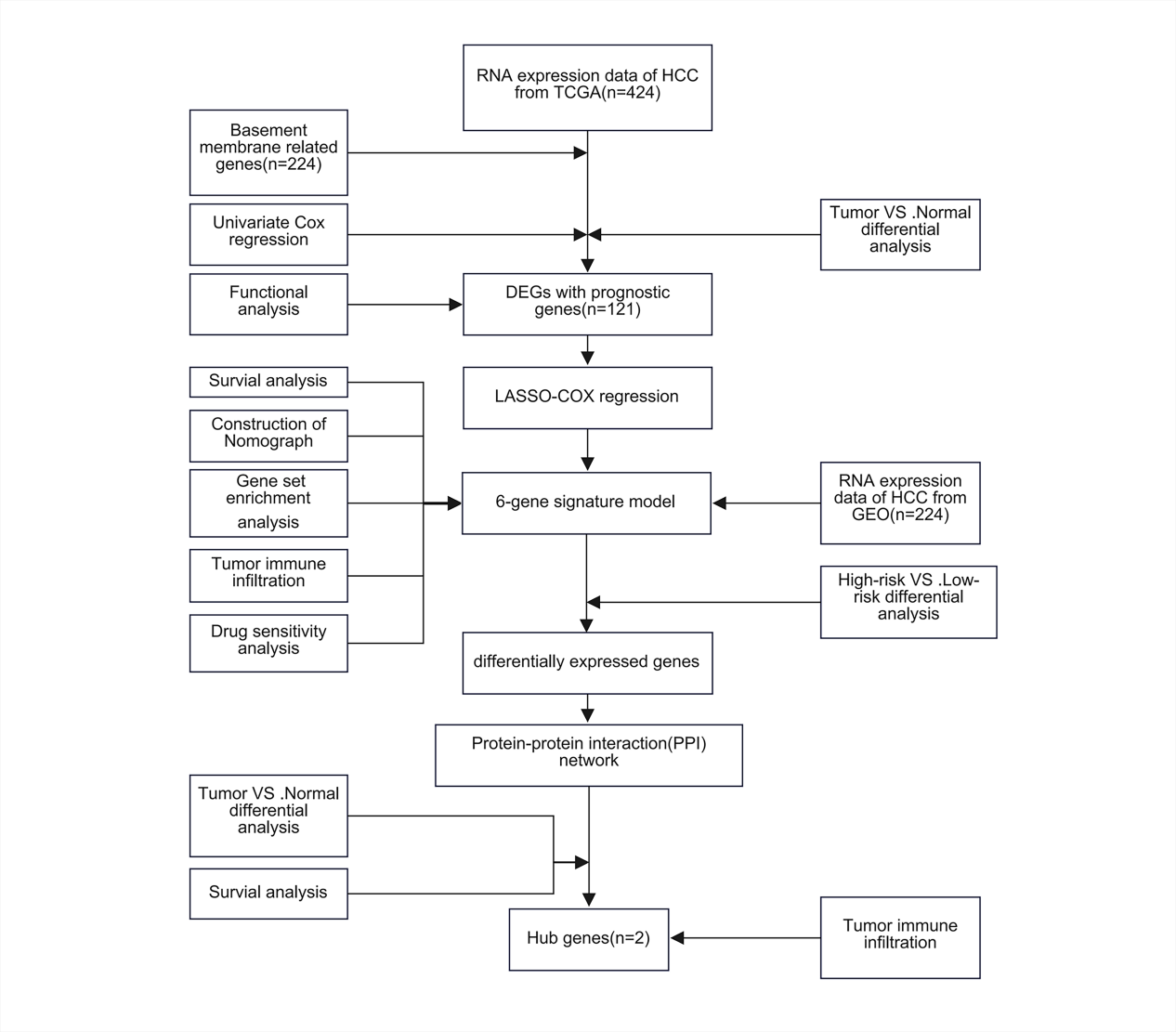


**Supplementary Figure 1**. The flow chart of the study


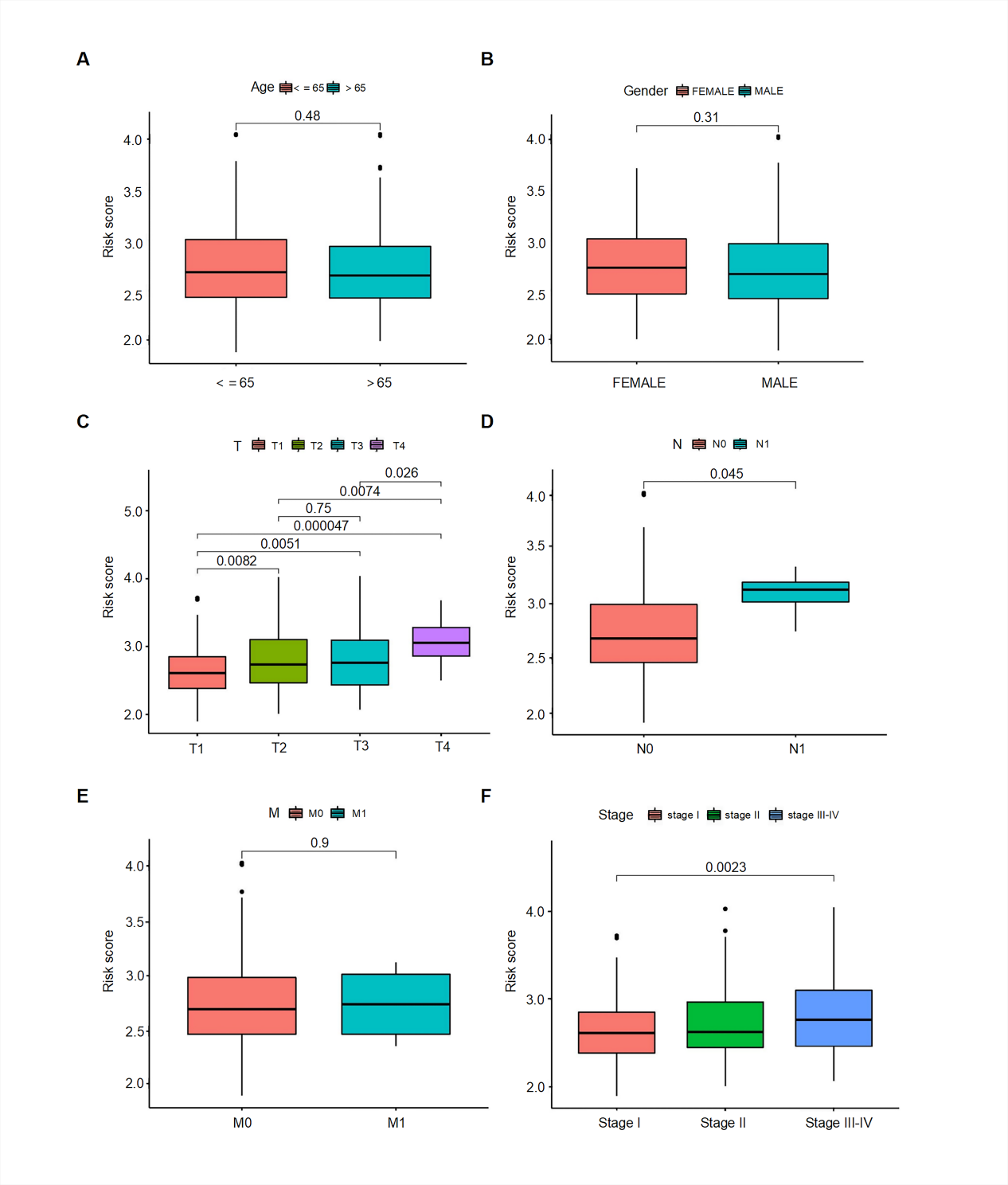


**Supplementary Figure 2**. (A-F) risk score and clinicopathological characteristics.


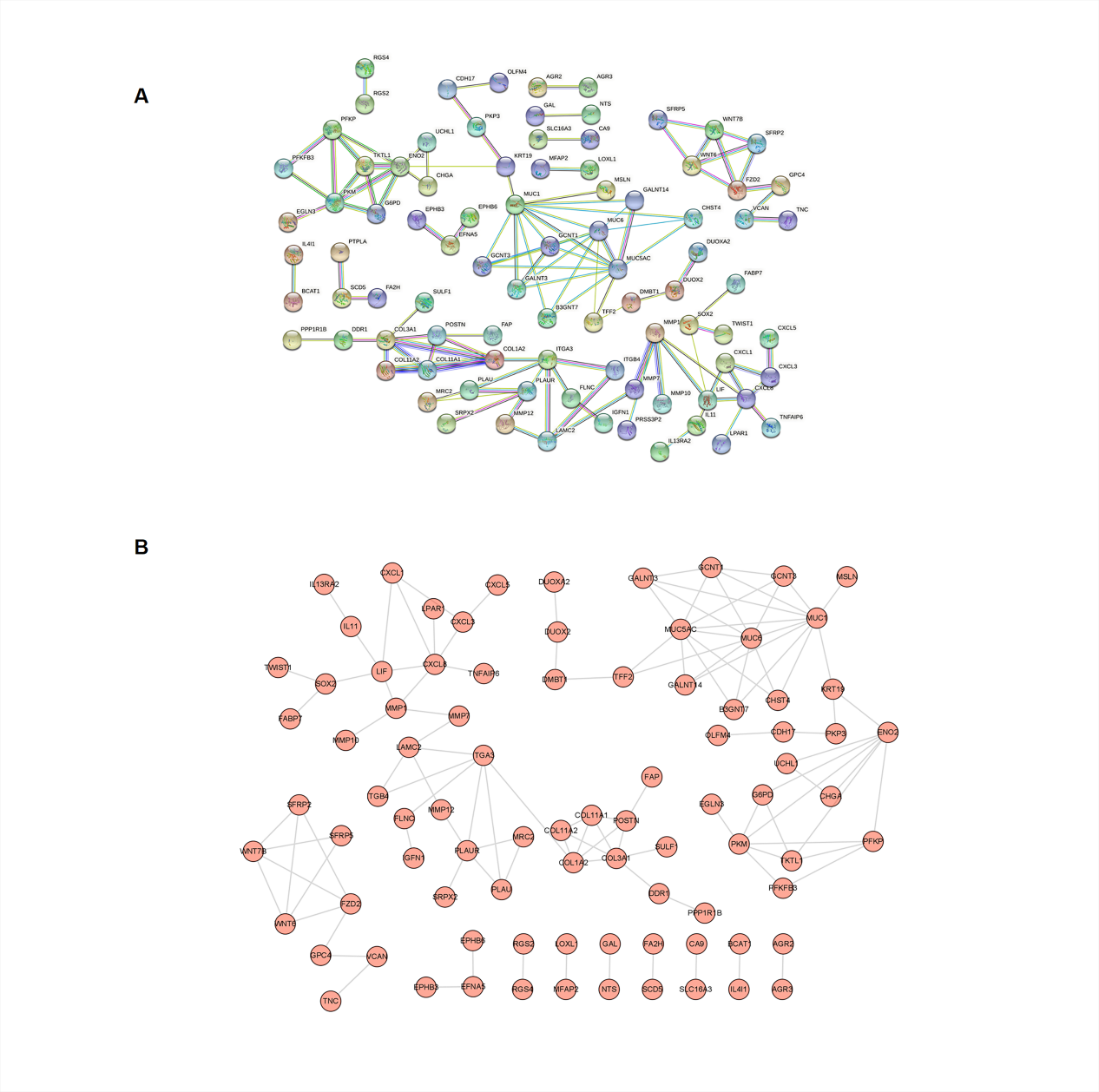


**Supplementary Figure 3**. (A) Protein-protein interaction (PPI) network of risk differential genes. (B) PPI network processed by Cytoscape.


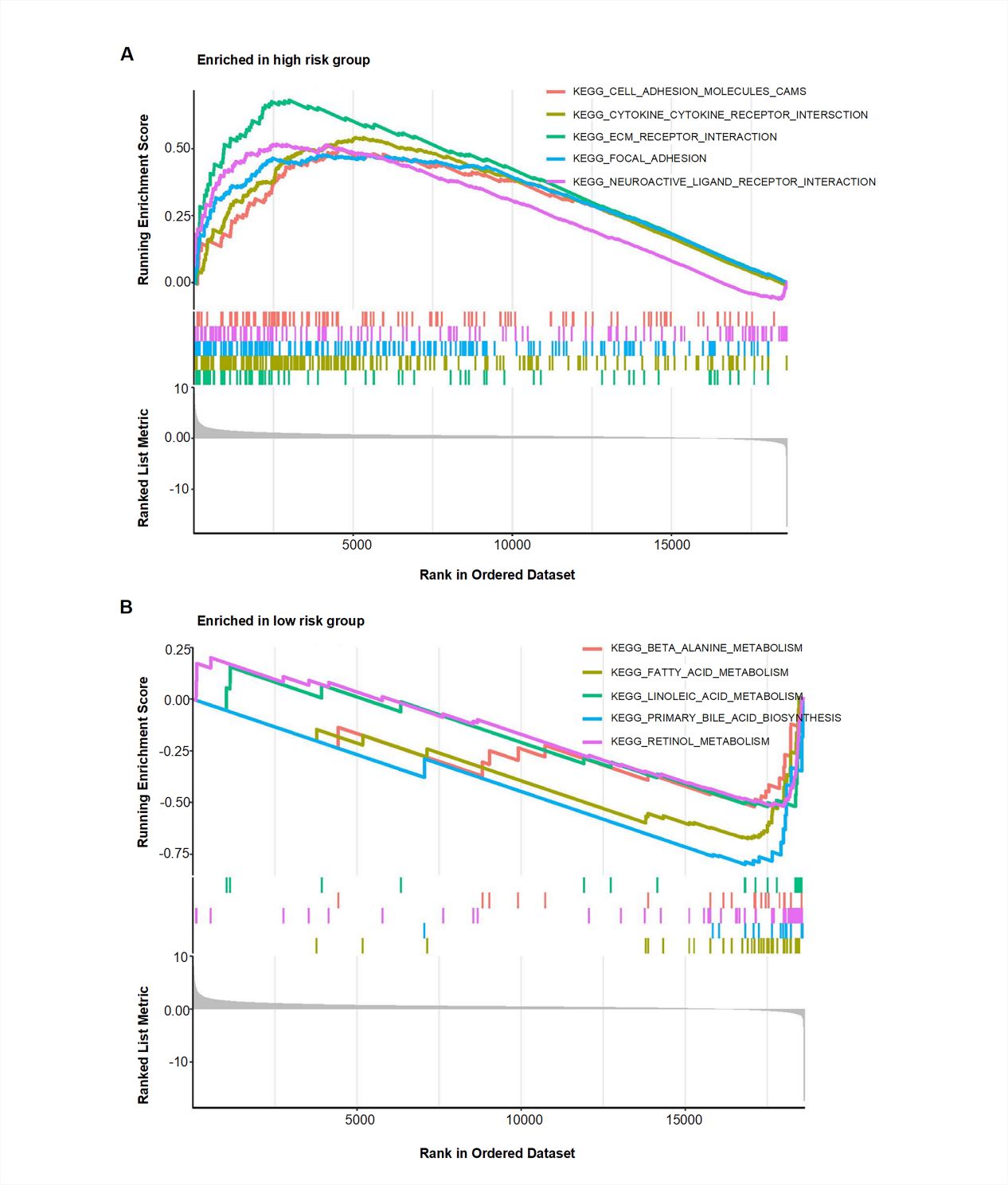


**Supplementary Figure 4**. (A) High-risk group GSEA analysis. (B) Low-risk group GSEA analysis.
